# The Influence of Chemical Surface Modification of Kenaf Fiber using Hydrogen Peroxide on the Mechanical Properties of Biodegradable Kenaf Fiber/Poly(Lactic Acid) Composites

**DOI:** 10.3390/molecules19032957

**Published:** 2014-03-07

**Authors:** Nur Inani Abdul Razak, Nor Azowa Ibrahim, Norhazlin Zainuddin, Marwah Rayung, Wan Zuhainis Saad

**Affiliations:** 1 Department of Chemistry, Faculty of Science, Universiti Putra Malaysia, 43400 Serdang, Selangor, Malaysia; E-Mails: norhazlin@upm.edu.my (N.Z.); marwahrayung@yahoo.com (M.R.); 2 Department of Microbiology, Faculty of Biotechnology and Biomolecular Sciences, Universiti Putra Malaysia, 43400 Serdang, Selangor, Malaysia; E-Mail: zuhainis@upm.edu.my

**Keywords:** poly(lactic acid), kenaf fiber, melt blending, hydrogen peroxide, composite

## Abstract

Bleaching treatment of kenaf fiber was performed in alkaline medium containing hydrogen peroxide solution maintained at pH 11 and 80 °C for 60 min. The bleached kenaf fiber was analyzed using Fourier Transform Infrared (FTIR) and X-ray Diffraction (XRD) analysis. The bleached kenaf fiber was then compounded with poly-(lactic acid) (PLA) via a melt blending method. The mechanical (tensile, flexural and impact) performance of the product was tested. The fiber treatment improved the mechanical properties of PLA/bleached kenaf fiber composites. Scanning electron micrograph (SEM) morphological analysis showed improvement of the interfacial adhesion between the fiber surface and polymer matrix.

## 1. Introduction

The utilization of plastics has become a vital feature in various commodities and industries. However, this vast consumption causes environmental pollution and accumulation in disposal systems as most conventional plastics are resistant to degradation. The growing environmental awareness about the non-biodegradability of plastic waste has triggered the search for biodegradable and renewable resources. Nowadays, the development of biodegradable polymers to overcome this problem has become one of the main areas of interest of researchers. One important biodegradable polymer is poly-(lactic acid), PLA, which originates from natural resources as it can be synthesized by ring-opening polymerization or condensation polymerization of lactic acid monomer obtained via fermentation of dextrose from starch feedstocks [1]. PLA also exhibits attractive properties such as high strength, superior modulus, biodegradable and ease of processing [2].

However, due to its relatively high cost, PLA cannot compete economically with conventional plastics. One way to reduce the cost is by combining PLA with inexpensive fillers such as natural fibers to produce a cost effective composite. Kenaf (*Hibiscus cannabinus*, Malvaceae) fiber has attracted much attention for this purpose because it offers both ecological and economic advantages. Kenaf can grow under a wide range of climate conditions to a height of more than 5–6 m in 6–8 months [3]. The cost of natural fiber is about $0.44 to $0.55 per kilogram, compared to more expensive synthetic fibers that cost $2.00 to $3.25 per kilogram [4]. Apart from that, kenaf fiber is suitable as a filler in composite materials because it is not abrasive during processing, and it is biodegradable, has low density and specific mechanical properties [5].

Due to the distinct properties of PLA and kenaf fiber, the combination of both materials causes poor interfacial adhesion because the natural fiber is hydrophilic whereas the polymer is hydrophobic [6]. Therefore, the surface of the fiber has to be treated in order to promote better interfacial adhesion. A lot of efforts have gone into modifying the properties of natural fiber with various chemical treatments such as alkali treatment (mercerization) [7,8], silane treatment [9,10] and acetylation [11]. Results from these studies showed that the treatment on the surface of the fiber can improve the mechanical properties of composites.

**Figure 1:**
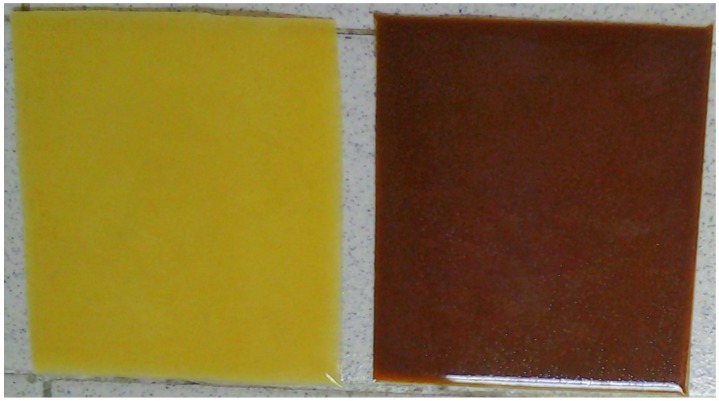
Image of bleached (**left**) and unbleached (**right**) kenaf fiber/PLA composite.

Natural fiber can be treated with hydrogen peroxide (H_2_O_2_) which is extensively used in textile industry [12,13]. However, only a few studies have reported about the effect of this treatment on the properties of polymeric composites. As a oxidizing bleaching agent, H_2_O_2_ causes discolouration of fiber. Thus, better physical appearance of composite can be obtained with incorporation of bleached fiber into the polymeric composite (Figure 1). Bleaching of H_2_O_2_ relies on the dissociation of perhydroxyl anion (HOO^−^, Equation (1)) which predominantly occurs under alkaline conditions:

H_2_O_2_ + OH^−^ → H_2_O + HOO^−^ (1)

In the case of lignocellulosic fiber, the colour of the fiber is due to the lignin component. It is believed that bleaching action takes place when the nucleophile (HOO^−^) attacks the carbonyls and conjugated carbonyl groups that comprise the fiber. Besides the improvement in physical appearance, fiber surface treatment can also enhance the mechanical performance of polymeric composites. Interestingly, improvement in both physical appearance and mechanical properties can be achieved in only one treatment procedure. In this study, the effect of hydrogen peroxide bleaching treatment on the mechanical properties of PLA/kenaf fiber composites was investigated. 

## 2. Results and Discussion

### 2.1. Fourier Transform Infrared (FTIR) Analysis of Untreated and Treated Fiber

**Figure 2:**
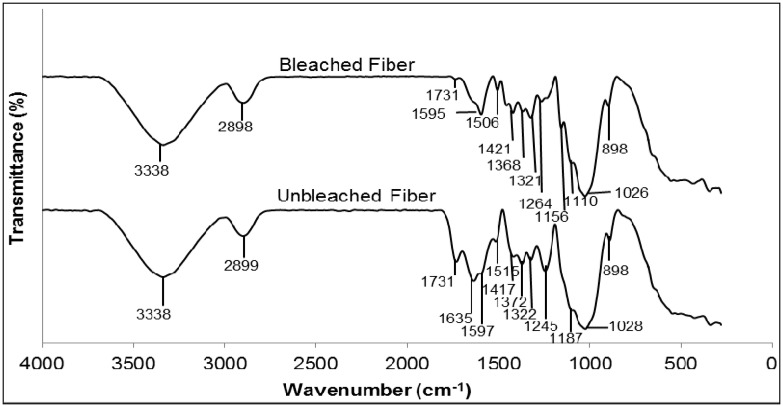
FTIR spectra of bleached and unbleached kenaf fiber.

FTIR analysis was conducted to study the characteristics of the kenaf fibers, before and after bleaching treatment with hydrogen peroxide. As depicted in Figure 2, the broad peak at 3338 cm^−1^ which appears in both spectra is attributed to the O–H frequency, whereas the peaks at 2898 cm^−1^ and 2899 cm^−1^ predominantly arise from C–H stretching [14]. Another peak at 1731 cm^−1^ corresponds to ester carbonyl vibrations from the acetyl, feruloyl and *p*-coumaryl groups in lignin. After bleaching treatment, there was a decrease in the intensity of these peaks, indicating that most of the lignin has been removed [15]. A stretching peak detected at 1635 cm^−1^ for unbleached fiber is attributed to the carbonyl group of the acetyl ester in hemicellulose and the carbonyl aldehyde in lignin [16]. The absence of this peak after the bleaching treatment may be due to the removal of lignin and hemicellulose. For bleached kenaf fiber, the disappearance of the vibration peak at 1,245 cm^−1^ that corresponds to C–O vibration is also attributed to the removal of lignin [17].

### 2.2. X-ray Diffraction Analysis of Unbleached and Bleached Kenaf Fiber

**Figure 3:**
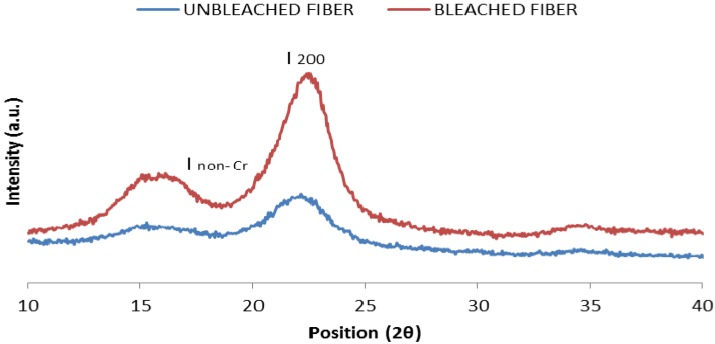
XRD patterns of unbleached and bleached fiber.

X-ray diffraction (XRD) analysis was conducted to compare the XRD patterns of bleached and unbleached kenaf fiber. The crystallinity index (CrI) of fiber was calculated using Equation (2) in the Experimental section, as reported by Segal *et al.* [18]*.* Figure 3 shows XRD diffractograms of unbleached and bleached fiber. The patterns exhibited an intense peak at around 2θ = 22° (I_200_) for both fibers. This peak corresponds to the crystallinity region in the fiber. The non-crystalline region of the fiber is shown by the valley between the peaks which was assigned as I_non-Cr_ at around 2θ = 18°. After treatment of the kenaf fiber, it can be seen that the peak at the 2θ position around 22° became more intense and narrower, which indicated a higher degree of crystallinity in the bleached fiber. Calculation of the crystallinity index showed that bleached fiber has a higher value compared to unbleached fiber, with values of 60.0% and 44.1%, respectively.. According to Janoobi *et al.* [14] the increase in crystallinity index can be attributed to the removal of lignin and hemicellulose after fiber treatment.

### 2.3. Effect of Bleaching Treatment of Fiber on Tensile Properties of PLA/Kenaf Fiber Composites

**Figure 4:**
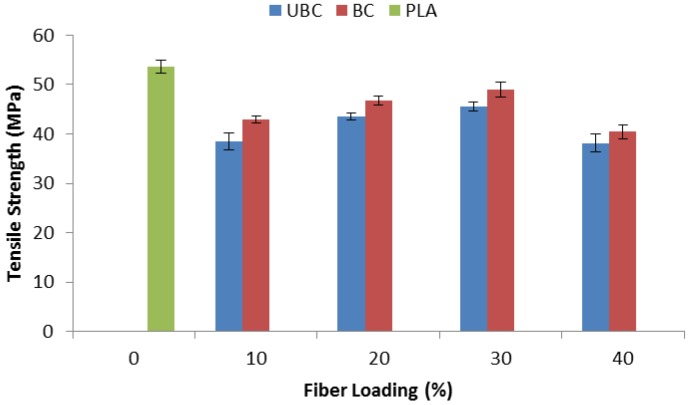
Tensile strength of pure PLA, UBC and BC composites.

Figure 4 shows the tensile properties of pure PLA, PLA/unbleached kenaf fiber composites (UBC) and PLA/bleached kenaf fiber composites (BC). The results clearly show that PLA exhibited better tensile strength compared to its composites and this finding was also in agreement with Oksman *et al.* [19]. With addition of 10 wt% of unbleached kenaf fiber, the tensile strength of PLA decreased from 53.6 MPa to 38.5 MPa. The incorporation of 10 wt% of bleached fiber to BC composite still exhibited a low tensile strength with 42.9 MPa. This decrease is probably due to an insufficient amount of kenaf fiber to impart strength to the composite. Improvement in tensile strength can be observed when fiber loading increased up to 30 wt%. At 30 wt% fiber content, the tensile strength of UBC and BC composite is 45.6 MPa and 48.9 MPa, respectively.

However, the addition of 40 wt% of fiber in the composite decreased the tensile strength. This may be due to insufficient matrix to wet out the fiber. BC composites showed better tensile strength at all fiber loadings compared to the corresponding UBC composites. Bleaching treatment of fiber caused the surface of the fiber to become rougher and created a better interlocking mechanism with the PLA matrix. This finding is supported by the SEM results which revealed better compatibility between fiber and matrix after bleaching treatment of kenaf fiber.

**Figure 5:**
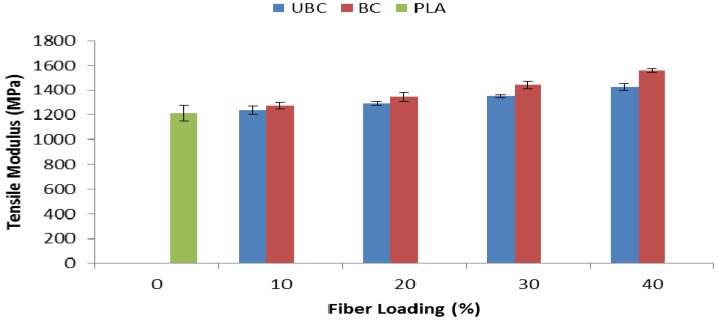
Tensile modulus of pure PLA, UBC and BC composites.

Figure 5 illustrates the tensile modulus of pure PLA, PLA/bleached kenaf fiber composites (BC) and PLA/unbleached kenaf fiber composites (UBC) at different fiber loadings. The results showed that incorporation of 10 wt% of kenaf fiber, either unbleached or bleached, caused an increment in the tensile modulus of the composites. Moreover, the value kept increasing as the fiber content reached 40 wt%. The tensile modulus of UBC and BC composite at 40 wt% was 1426.1 MPa and 1557.6 MPa, respectively. As we increase the fiber content in the composite, it restricts the mobility of the matrix, consequently, the composites became stiffer and the tensile modulus increased. Additionally, it is also evident that BC composites exhibited better tensile modulus at each fiber loading compared to UBC composites. Improvement in interfacial interaction between the bleached kenaf fiber and its matrix, PLA, is reflected in the better tensile modulus properties of BC composites.

**Figure 6:**
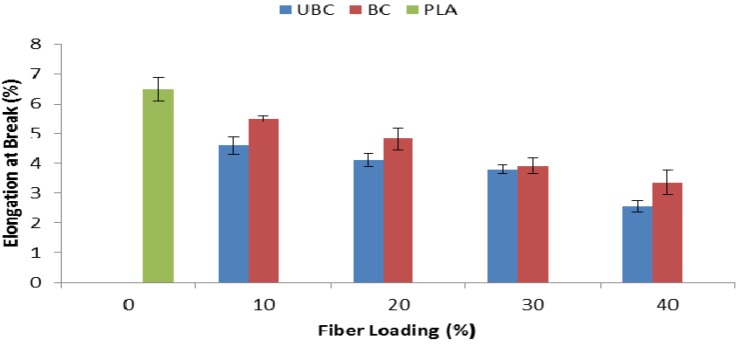
Elongation at break of pure PLA, UBC and BC composites.

Elongation at break properties of pure PLA, PLA/unbleached kenaf fiber composites (UBC) and PLA/bleached kenaf fiber composites (BC) are shown in Figure 6. In general, the elongation at break properties of the composites decreased when 10 wt% of kenaf fiber was added and the value was gradually reduced with more addition of kenaf fiber. As we increased the fiber content, the composites became more rigid and the fiber restricted the stretching of composites [20]. Therefore, the elongation at break decreased due to the low deformation behavior of the composites. Apart from that, it can be observed that elongation at break properties of BC composites is higher compared to UBC composites.

### 2.4. Effects of Bleaching Treatment of Fiber on Flexural Properties of PLA/Kenaf Fiber Composites

**Figure 7:**
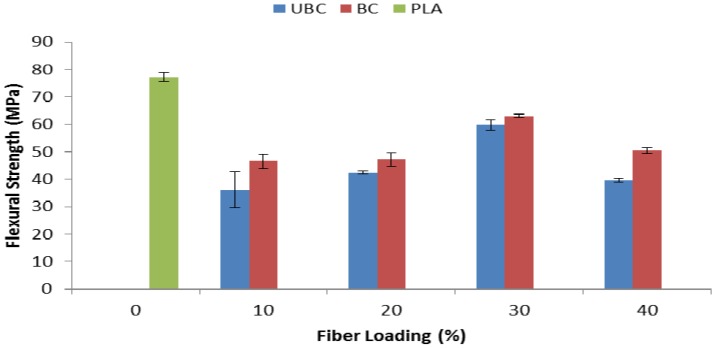
Flexural strength of pure PLA, UBC and BC composites.

Flexural tests were also performed to gain a better understanding of the mechanical behavior of the composites. The flexural strength properties of pure PLA, UBC and BC composites at various fiber compositions are represented in Figure 7. Like in the case of the tensile strength, the incorporation of kenaf fiber led to lower flexural strength of the composites compared to pure PLA. As the fiber content increased from 10 to 30 wt%, both composites, UBC and BC, showed an increment in the flexural strength. However, the value decreased at 40 wt% of unbleached/bleached kenaf fiber. Like the tensile strength, the flexural strength trend showed that BC composites have higher flexural strength at each fiber loading than the corresponding UBC composites.

**Figure 8:**
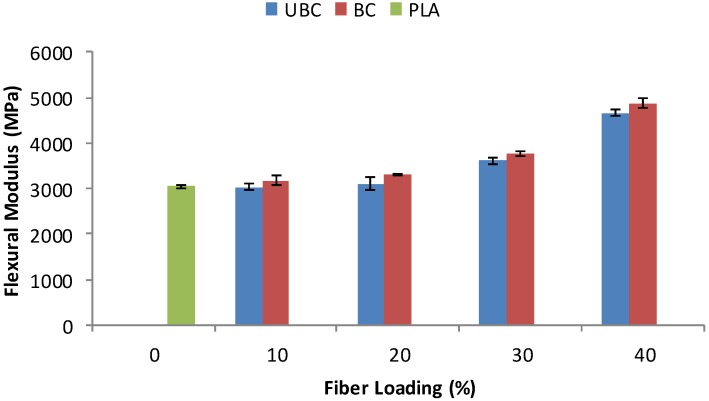
Flexural modulus of pure PLA, UBC and BC composites.

Flexural modulus properties of pure PLA and its composites are shown in Figure 8. Flexural modulus showed a similar trend as tensile modulus. When the fiber content increased from 10 to 40 wt%, the flexural modulus of both UBC and BC composites increased. In addition, the flexural modulus of BC composite is higher compared to UBC composite at each fiber loading. A study by Huda *et al.* [7] also demonstrated the improvement in flexural modulus of the PLA/kenaf fiber composites when the fiber was treated with chemical treatment. They claimed that the increase is influenced by the good compatibility between fiber and matrix.

### 2.5. Effects of Bleaching Treatment of Fiber on Impact Properties

**Figure 9:**
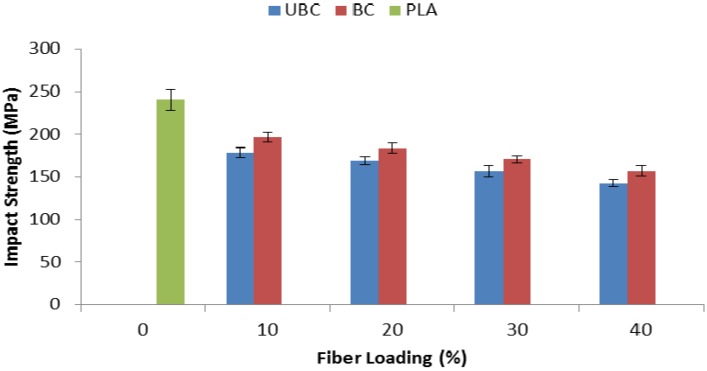
Impact strength of pure PLA, UBC and BC composites.

Figure 9 shows the Izod impact strength for PLA, UBC and BC composites at various fiber loadings. The results revealed that the use of kenaf fiber resulted in a reduction of the impact strength of the composites. Moreover, a continuous decrease in impact strength also can be observed as the fiber composition was increased from 10 to 40 wt%. Nevertheless, when compared to UBC composites, BC composites showed improvement in impact strength. According to Devi *et al.* [21], the energy absorbing mechanism depends on the interfacial interaction between fiber and matrix. Therefore, it is believed that the increase is obtained due to the improvement in fiber-matrix adhesion of BC composites after the fiber is bleached with hydrogen peroxide. For UBC composites, poor fiber-matrix adhesion initiated a crack in the inner part of the composite which required less energy to break the sample [22].

### 2.6. SEM Morphology of Fiber and Composites

**Figure 10:**
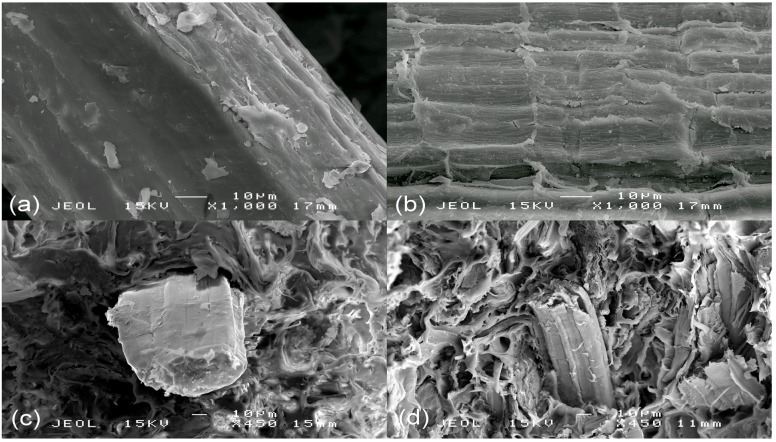
SEM images of (**a**) untreated fiber, (**b**) treated fiber (**c**) UBC composite (**d**) BC composite.

In order to study the increase in tensile strength, the tensile fracture morphology of both UBC and BC composites at 30 wt % of fiber loading were analyzed using the Scanning Electron Microscopy (SEM) technique. Figure 10a,b shows the surface morphology of unbleached and bleached fiber, respectively. There are small particles which adhered to the fiber (might be waxes) that disappeared after bleaching treatment. Furthermore, the surface morphology of bleached kenaf fiber also became rougher and textured. A rough fiber surface can create good interlocking with the matrix surface and give good PLA-kenaf fiber adhesion. This can be proved from the tensile fracture morphology of BC composite (Figure 10d) which shows close gaps between the PLA matrix and kenaf fiber. On the other hand, poor interfacial adhesion can be seen for UBC composite due to the wide bonding gap between fiber and PLA (Figure 10c). It is important to note that improvement in mechanical properties of the polymeric composites can result from good compatibility between fiber and matrix [23].

## 3. Experimental

### 3.1. Materials

PLA pellets (Grade: 4060D) with density of 1.24 g/cm^3^ was purchased from Nature Works LLC, (Minnetonka, MN, USA). Kenaf fiber kindly supplied by the Institute of Tropical Forestry and Forest Products (INTROP), UPM, was sieved to 300 µm before use. The materials were dried in an oven at 60 °C for 24 h prior to compounding to minimize moisture content. Hydrogen peroxide solution (30% *v/v*) and sodium hydroxide (NaOH) in pellets formed were bought from R&M Chemicals (Essex, UK).

### 3.2. Kenaf Fiber Bleaching Treatment

The kenaf fiber was immersed in a solution containing hydrogen peroxide (5% *v/v*) for 60 min at pH 11 and the temperature was maintained at 80 °C in water bath. Sodium hydroxide (NaOH) with concentration of 0.5 M was used to adjust the pH until the solution reached pH 11. The fiber was then thoroughly washed with distilled water and dried in an oven at 60 °C for 48 h.

### 3.3. Preparation of Composites

Poly(lactic acid) pellets were compounded with varying amounts of bleached kenaf fiber (10, 20, 30 and 40 wt%). Compounding was performed at 160 °C with speed of 50 rpm for 15 min using a HAAKE polydrive internal mixer (Karlsruhe, Germany). For comparison purposes, composites of PLA/unbleached kenaf fiber were also prepared with the same fiber composition. After compounding, the composites were compressed into sheets using a hydraulic hot-press.

### 3.4. Characterization

#### 3.4.1. Fourier Transforms Infrared (FTIR) Analysis

The FTIR analysis was conducted by using a Fourier Transform Infrared (FTIR) spectrometer (model spectrum 100, Perkin Elmer, Waltham, MA, USA) with the diamond attenuated total reflectance (ATR) technique. The FTIR test was performed over the wavenumber range of 280 to 4000 cm^−1^.

#### 3.4.2. X-ray Diffraction (XRD) Analysis

X-ray diffraction analysis was carried out by using a Shimadzu XRD 6000 X-ray diffractometer (Tokyo, Japan) with CuKα radiation (λ = 1.542 Å) operated at 30 kV and 30 mA. Data were collected within the range of scattering angles (2θ) of 10° to 40° at room temperature. The crystallinity index (CrI) was calculated as below:

CrI (%) = [ (I_200_ − I_Cr-non_)/I_200_] × 100 (2)

where I_200_ represents the peak intensity of the crystalline region, whereas I_Cr-non_ denotes the non-crystalline region.

#### 3.4.3. Tensile Tests

The tensile test was performed at ambient temperature by using an Instron Universal Testing Machine (Model 4302 Series IX, Instron, Norwood, MA, USA) based on ASTM D638. Dumbbell shape specimens were cut from 1.00 mm sample sheet of each composition. The test was carried out at a constant crosshead speed of 5 mm/min and load cell of 1 kN.

#### 3.4.4. Flexural Tests

Flexural test was conducted using an Instron Universal Testing Machine (Model 4302 Series IX) equipped with a 1 kN load cell, according to ASTM D790. Flexural strength and flexural modulus were obtained at constant crosshead speed of 3 mm/min.

#### 3.4.5. Izod Impact Test

The impact strength measurement was determined according to ASTM D256, by using an Izod Impact Tester (International Equipment, Mumbai, India) which equipped with a 453 g pendulum. Impact strength was calculated by dividing energy (J) with the thickness of specimen (m).

#### 3.4.6. Scanning Electron Microscopy (SEM)

The surface morphology of the tensile fractured composites was examined by a JEOL Scanning Electron Microscope (JSM6400, JEOL Ltd., Tokyo, Japan) with an acceleration voltage of 20 kV. Samples were coated with gold to avoid electron charging effects during examination.

## 4. Conclusions

Modification of the surface of kenaf fiber was done by bleaching with hydrogen peroxide under alkaline conditions. This treatment caused an increase in crystallinity index and surface roughness of the kenaf fiber due to the removal of lignin and hemicellulose after the bleaching treatment. The increase in surface roughness of the kenaf fiber created good interlocking with the PLA matrix, hence, it altered the interfacial adhesion between PLA and fiber. As a result, the mechanical properties of PLA/bleached kenaf fiber composites (BC) are modified.
